# The Continuing Care Model of Substance Use Treatment: What Works, and When Is “Enough,” “Enough?”

**DOI:** 10.1155/2014/692423

**Published:** 2014-03-27

**Authors:** Steven L. Proctor, Philip L. Herschman

**Affiliations:** ^1^Department of Psychology, Louisiana State University, 236 Audubon Hall, Baton Rouge, LA 70803, USA; ^2^CRC Health Group Inc., 6185 Paseo Del Norte, Suite 150, Carlsbad, CA 92011, USA

## Abstract

There is little disagreement in the substance use treatment literature regarding the conceptualization of substance dependence as a cyclic, chronic condition consisting of alternating episodes of treatment and subsequent relapse. Likewise, substance use treatment efforts are increasingly being contextualized within a similar disease management framework, much like that of other chronic medical conditions (diabetes, hypertension, etc.). As such, substance use treatment has generally been viewed as a process comprised of two phases. Theoretically, the incorporation of some form of lower intensity continuing care services delivered in the context of outpatient treatment after the primary treatment phase (e.g., residential) appears to be a likely requisite if all stakeholders aspire to successful long-term clinical outcomes. Thus, the overarching objective of any continuing care model should be to sustain treatment gains attained in the primary phase in an effort to ultimately prevent relapse. Given the extant treatment literature clearly supports the contention that treatment is superior to no treatment, and longer lengths of stay is associated with a variety of positive outcomes, the more prudent question appears to be not whether treatment works, but rather what are the specific programmatic elements (e.g., duration, intensity) that comprise an adequate continuing care model. Generally speaking, it appears that the duration of continuing care should extend for a minimum of 3 to 6 months. However, continuing care over a protracted period of up to 12 months appears to be essential if a reasonable expectation of robust recovery is desired. Limitations of prior work and implications for routine clinical practice are also discussed.

## 1. Introduction

Substance use and perhaps, more importantly, substance use disorders (SUD) remain important public health and safety concerns in the USA. Recent findings from the National Survey on Drug Use and Health indicate that an estimated 20.6 million (8.0%) persons aged 12 years or older in the US general population meet current* Diagnostic and Statistical Manual of Mental Disorders, Fourth Edition* (DSM-IV; [1]), criteria for a SUD (i.e., dependence or abuse) [2]. Of these, over three-fourths were classified with a past 12-month alcohol use disorder (AUD), and marijuana was the specific illicit drug with the highest level of past year dependence or abuse, followed by prescription pain relievers and cocaine, respectively [2]. Substance use and SUDs have also been associated with a variety of untoward outcomes, including hospitalization, impaired driving and motor vehicle accidents, increased vulnerability to other serious medical conditions or infections, additional substance use and psychiatric comorbidity, criminal activity, cognitive impairment, and mortality [2–10].

With respect to health care utilization, alcohol and illicit drug use both pose a significant burden on US hospital emergency departments, with rates of substance-related visits continuing to increase annually [11, 12]. In fact, some estimates indicate that as many as 7.6 million emergency department visits are attributable to alcohol each year, accounting for nearly 1 in 10 of all US emergency department visits annually [13]. Recent data from the Drug Abuse Warning Network, a nationally representative public health surveillance system, also underscore the increasing involvement of illicit drugs in emergency department visits with 2.3 million persons presenting with a problem attributable to illicit drug misuse in 2010 [9]. Individuals with demonstrated substance dependence or problematic use have also been found to utilize health care services at a higher rate than adults without substance use problems [14, 15]. Although medical care utilization is higher among inpatient than outpatient substance use treatment populations [16], individuals with a SUD, irrespective of level of care, have been found to account for greater health care expenditures than adults without a SUD in the US general population [17]. Thus, health care utilization and associated costs appear to be a function of substance use severity.

The harmful effects of substance use and SUDs are also of concern in the workplace. National estimates suggest that workplace alcohol use and impairment directly affect an estimated 15% (or 19.2 million) of employed adults in the USA [18]. Specifically, 9.23% reported working while experiencing the lingering effects of heavy alcohol use from the night prior to work (i.e., working with a hangover), 7.06% consumed alcohol during the workday, while on the job, 1.83% consumed alcohol within two hours of reporting to work, and 1.68% worked while being under the influence of alcohol. Alcohol-dependent employees and those that engage in heavy alcohol consumption have also been found to evince high rates of absenteeism, poor work performance (e.g., arriving to work late, leaving work early, completing less work), and other factors that are detrimental to overall productivity [19–21]. Similar to workplace alcohol use and impairment, an estimated 14% (or 17.7 million) of the US workforce reported past 12-month illicit drug use, with marijuana constituting the most commonly reported illicit drug used [22]. However, illicit drug use in the workplace, or using while being “on the job” (i.e., during lunch or other designated breaks from work), is much lower relative to alcohol, with 3.1% of employed adults reporting use of any illicit drug during normal work hours. Furthermore, 2.71% reported using an illicit drug within two hours of reporting to work, and 1.72% reported using while performing one's job. Together, these findings suggest that alcohol and illicit drug use both represent a notable problem among the US workforce and have the potential to impact work productivity.

Also noteworthy is the immense economic burden posed by SUDs. In fact, the estimated economic cost of alcohol dependence and abuse in the USA was $185 billion in 1998 alone [23]. This figure also reflects the estimated total loss of earnings attributable to AUDs. Specifically, the US businesses can expect annual financial losses in excess of $97.7 billion due to employees with alcohol dependence or abuse, primarily as a result of economic productivity lost due to alcohol-related illness, injury, and crime [23]. If one considers the loss of projected earnings due to premature alcohol-related mortality (e.g., motor vehicle accidents), AUDs account for an estimated $134 billion in financial losses. Regarding the total health care expenditures, AUDs have been found responsible for an estimated $18.8 billion in spending. Interestingly, the total estimated cost of illicit drug dependence and abuse is quite comparable to that of AUDs, which was approximately $181 billion in 2002 [24]. In particular, drug dependence and abuse accounted for an estimated $129 billion in productivity losses and an estimated $15.8 billion in health care related costs (e.g., hospital and ambulatory care).

In sum, high rates of SUDs precipitating increased involvement in health care utilization, coupled with the persistent and pervasive problem of substance use and related impairment among the US workforce, suggest that SUDs are associated with considerable negative outcomes. Whether an individual is under the influence of a substance during normal work hours, unable to attend work due to the effects of their substance use, incarcerated for the commitment of a substance-related offense, or hospitalized as the result of substance-related injuries or violence, their incapacity amounts to a loss in workplace productivity and ultimately substantial financial losses. SUDs also present demonstrable medical, social, and psychological costs. In light of the range of impairment and adverse consequences associated with SUDs to the individual, employers, and the US society at large, the need for an effective solution remains of paramount importance. One potential option to offset the general health care, employment, and societal costs is SUD treatment.

## 2. Levels of Substance Use Treatment

The treatment of SUDs involves varying levels of care and may include any one or combination of a number of psychosocial approaches. In the sections to follow, a general overview of the various levels of SUD treatment is presented. Although relatively short forms of treatment, particularly brief motivational interventions consisting of a single 10- to 50-minute session or two 50-minute sessions, have been found efficacious in the treatment of a substance use and related problems among “high-risk” substance users (i.e., individuals meeting DSM-IV criteria for substance abuse or “mild” substance dependence) in a variety of setting, the following sections will focus exclusively on the standard levels of care for individuals meeting DSM-IV criteria for substance dependence or moderate to severe SUD as specified in the* Diagnostic and Statistical Manual of Mental Disorders, Fifth Edition* (DSM-5; [25]).

Substance use treatment has generally been conceptualized as a process comprised of two phases. With respect to the initial or primary phase of the treatment of substance dependence, detoxification, residential, and in some cases, intensive-outpatient treatment (IOT), or, in even fewer cases, standard outpatient treatment services may be indicated. IOT is used here to include partial hospitalization (PHP, minimum of 5 days per week and 20 total hours of direct service per week) and intensive outpatient (IOP, minimum of 3 days per week and 9 hours of service per week). Depending on the level of care received during the primary phase of treatment, the second phase typically involves some form of less intensive and tapered care (e.g., PHP, community-based self-help/support groups), which can range in duration from a few weeks to up to several years.

### 2.1. General Summary of SUD Treatment

Length of stay in substance use treatment has long been considered as one of the most reliable predictors of posttreatment outcomes by way of several previous large-scale, multisite studies of treatment effectiveness in the USA. [26–29]. In the latest U.S. national treatment evaluation project, the Drug Abuse Treatment Outcomes Study (DATOS), a total of 10,010 patients admitted to 96 programs from 11 cities participated in the project from 1991 to 1993 (for more information regarding methodology and research design for DATOS see [30]). The sample included patients admitted to publically funded and private long-term residential programs, short-term inpatient programs, outpatient treatment programs, and outpatient methadone maintenance programs. Overall, the initial DATOS evaluation project and subsequent family of studies replicated prior work in that longer length of stays were associated with better follow-up outcomes [31–33]. This general conclusion was consistently found despite considerable variation in how the programs operated, the populations treated, their success in engaging and retaining patients in treatment, and the specific services delivered.

Specifically, patients receiving 3 months or more of treatment in long-term residential and outpatient treatment demonstrated significantly better outcomes with respect to lower rates of illicit drug use and improvements in several additional areas of behavioral functioning (e.g., employment, criminality) at the 12-month follow-up relative to patients with treatment durations of less than 3 months [31–33]. Regarding outpatient methadone maintenance services, however, it was not until patients had remained in treatment for 12 months or longer that they demonstrated significantly greater reductions in illicit drug use behaviors at follow-up than patients who dropped out of treatment prior to 12 months [32, 33]. Moreover, the odds of weekly or more frequent use of cocaine, alcohol, and marijuana among patients who stayed in residential treatment for 3–6 months decreased as patients remained in treatment for 6–12 months and again at 12 months or longer [31]. A similar pattern of substance use was found for patients staying in outpatient treatment for 6–12 months, compared to patients staying for 3–6 months. Overall, the findings revealed a progressively greater reduction in the likelihood of substance use after long-term residential and outpatient treatment as length of stays increased.

It is important to note, however, that some evidence, albeit limited, suggests that longer length of stays may not be justified during the primary phase of treatment for certain patients receiving care in the context of a residential substance use treatment program [34, 35]. Harris et al. [34] tested the generally accepted hypothesis that longer treatment stays were associated with better clinical outcomes among a sample of Veterans presenting for residential substance use treatment. Patients were recruited from 28 randomly selected Veterans Health Administration Substance Abuse Residential Rehabilitation Treatment Programs (SARRTPs). Main outcomes included Addiction Severity Index (ASI; [36]) Alcohol and Drug composite scores. Results revealed that patients receiving residential treatment for an average of 90 days demonstrated significantly less improvement with respect to ASI alcohol composite scores than patients with an average length of stay of both 15 to 30 and 31 to 45 days. Limitations included, most notably, a relatively high attrition rate, which resulted in only slightly more than half of the total sample completing the follow-up outcome assessment, as well as the use of retrospective self-report measures of substance use. In the instance of missing outcome data for a sizeable number of patients (i.e., 40.4%), it is possible that more complete follow-up data might have altered the findings in favor of longer length of treatment stays given that the poor retention rate may be attributed to high rates of treatment dropout; although additional follow-up data have the potential to reinforce the observed findings as well.

In sum, evidence in the form of various clinical outcomes from several randomized controlled trials and systematic reviews of the SUD treatment literature clearly demonstrates that, irrespective of treatment modality, treatment affords improvements for the majority of patients and is undoubtedly better than no treatment. Longer length of treatment stays has also been associated with more favorable clinical outcomes [29, 31–33]. Finally, although more recent meta-analytic studies have tempered findings from earlier work, there does not appear to be consistent support for residential treatment over outpatient treatment placement for most substance-dependent patients [37]. Thus, extant research has amassed to support the contention that substance-dependent patients are likely to benefit from treatment despite differences in the specific theoretical orientation of the clinician, professional background and personal substance use history of the clinician, and in many instances, level of care received [38–45].

## 3. Continuing Care Model of Substance Use Treatment

Successful completion of the initial phase of substance use treatment, defined by the authors of the latest American Society of Addiction Medicine's Patient Placement Criteria [46] as the resolution of the problem(s) that justified admission to the patient's current level of care as indicated by achievement of the specific goals articulated in their individualized treatment plan, is generally followed by some form of continuing care, in which patients receive treatment of a lower intensity. For example, a patient favorably discharged from residential treatment may begin to receive IOT services. Should the initial treatment phase consist of IOT care, however, the patient would receive a lower intensity outpatient care following completion (i.e., standard outpatient treatment services). In general, substance-dependent patients begin with medically managed residential treatment, step down to IOT care, and then move to standard outpatient treatment care. Community-based self-help groups, such as Alcoholics Anonymous (AA; [47]) or Narcotics Anonymous (NA; [48]), also represent a common form of continuing care. Thus, although the initial treatment episode and accompanying level of care may vary based on a number of factors including, most notably, the patient's substance use severity (e.g., quantity and frequency of substance use, presence or absence of withdrawal symptoms), an important characteristic of any continuing care model is that the patient subsequently receive some form of lower intensity treatment following completion of the primary phase of treatment.

The sections to follow will first provide a definition of which treatment services constitute the concept of “continuing care,” followed by a selective review of the treatment outcomes research specific to continuing care treatment models. It is important to note that a comprehensive review of all available treatment services that may be considered as some form of continuing care treatment and their effectiveness is clearly beyond the scope of the present report. Similarly, participation in some form of continuing care following completion of the primary phase of treatment has been shown to offset the economic costs associated with service delivery and positively impact a variety of outcomes beyond those specific to substance use [16, 49–51]. However, an exhaustive discussion of the additional outcomes that may be expected from continuing care (e.g., health care utilization, occupational functioning and various indicators of work performance) will not be presented given the focused objectives of the present report—to review the continuing care treatment outcomes literature and identify the key programmatic elements associated with an effective continuing care model. Studies selected for inclusion in the present review were identified though several resources. Literature searches of the PsycINFO, PsycARTICLES, Psychology and Behavioral Sciences Collection, and MEDLINE databases were performed using various combinations of the subject terms: “substance dependence,” “substance use,” “alcohol dependence,” “alcoholism,” “drug use,” “treatment,” “continuing care,” “aftercare,” “stepped care,” “step-down care,” “continuum of care,” and “disease management.” The reference lists of identified studies and prior review articles were also examined for additional relevant sources.

### 3.1. Continuing Care Definition

Many terms associated with the concept of the division of treatment services into phases have been used interchangeably in the substance use treatment literature [52]. For instance, “aftercare” and “step-down care” have often been used to denote relatively brief, less intensive treatments beyond the primary, more intensive phase of care. That is, the treatment literature occasionally uses these two terms when referring to traditional outpatient treatment that follows residential or IOT, while other references of these terms involve discussing patients' participation in community-based self-help or support groups (e.g., AA/NA) after “formal” treatment is completed. “Disease management,” a related term, implies the use of some form of protracted therapeutic contact in an effort to manage the symptoms and impairment associated with substance dependence. Conversely, “stepped care” and “continuum of care” refer to entire systems or a model of treatment delivery in which the intensity of care is commensurate with the patient's response to treatment (i.e., patients are moved between various levels of care differing in intensity as their symptoms improve or worsen). Finally, “continuing care” also refers to treatments provided after the initial phase of care but has, historically, often implied more long-term treatment. However, continuing care has also been used to designate the community-based self-help groups available following formal treatment completion. Thus, for the purposes of the present report, the provision of any form of treatment services following the initial phase of treatment, irrespective of duration or level of care, is defined as “continuing care.”

Despite the apparent lack of consistency regarding terminology, there is little disagreement in the substance use treatment literature regarding the conceptualization of substance dependence as a cyclic, chronic condition consisting of alternating episodes of treatment and subsequent relapse [53, 54]. Likewise, substance use treatment efforts are increasingly being viewed and contextualized within a similar disease management framework, much like that of other chronic medical conditions such as diabetes and hypertension [55, 56]. Theoretically, the incorporation of some form of lower intensity continuing care services delivered in the context of outpatient treatment after the primary treatment phase (e.g., residential) appears to be a likely requisite if all stakeholders aspire to long-term successful clinical outcomes. In other words, the overarching objective of any continuing care model should be to sustain treatment gains attained in the primary phase in an effort to ultimately prevent relapse. Thus, given the extant treatment literature clearly supports the contention that treatment is superior to no treatment, and longer length of stays is associated with a variety of positive outcomes, the more prudent question appears to be not whether treatment works but rather what are the specific programmatic elements (e.g., duration, intensity) that comprise an adequate continuing care model and have the potential to result in the most favorable long-term clinical outcomes.

### 3.2. Continuing Care Treatment Outcomes

To begin our review of the continuing care treatment outcomes literature it is important to highlight the seminal works by Vannicelli [57] and Costello [58] regarding the impact of continuing care on the clinical outcomes of alcohol-dependent patients. Vannicelli followed 100 male and female patients for 6 months following discharge from a 4- to 6-week residential treatment program. The residential treatment program offered a formal 5-week aftercare program (beginning 1 to 2 weeks before discharge and continuing through 3 weeks after discharge) delivered in group format, as well as a menu of both open and closed group therapy options that patients were encouraged to attend following discharge (e.g., women's group, medication group, religious resources group). Outcome variables included self-reported substance use and a total score derived from a measure of alcohol use frequency and related impairment (i.e., social, medical, employment, and marital problems) at 3- and 6-month intervals. Several positive correlations were found between outcome and various indices of formal aftercare participation at both the 3- and 6-month follow-up, including those involving time since last drink with total number of meetings attended during the first 5 weeks after discharge, total number of meetings attended during the first 3 months, number of meetings of open aftercare groups attended, number of meetings of the 5-week aftercare package attended, and number of different kinds of groups attended. A similar pattern of correlations was found between alcohol-related problems and the six aforementioned aftercare variables. Overall, continuing care participation in the first 3 months following discharge from residential treatment appears to be significantly related to fewer days since last drink and lower levels of alcohol-related impairment at both 3 and 6 months.

Costello [58] later extended the findings presented by Vannicelli [57] by accounting for relevant patient prognostic characteristics and extending the duration of the follow-up interval. Costello followed 37 Caucasian male patients for 24 months following discharge from a 6-week residential treatment program (i.e., inpatient therapeutic community located within a hospital). Patient prognostic indicators included measures of social stability at admission to the treatment program and behavioral adjustment throughout the entirety of their treatment stay. Continuing care was defined and measured simply as the total number of subsequent visits to the same program at which the patients completed residential treatment to receive Antabuse (a popular prescription medication at the time commonly used as part of a treatment plan to reduce the desire to drink by producing an immediate and severe negative reaction to alcohol intake) or individual counseling, and attendance at “social gatherings.” The primary outcomes consisted of patients' scores on a measure of social adjustment at 12 and 24 months covering six parameters (i.e., drinking status, employment status, residential stability, general health status, family or other interpersonal relationships, and recreational and social activities) and a summed score representing patients' overall postdischarge adjustment. Results revealed that attendance at some form of continuing care improved the prognosis of alcohol-dependent patients at 12 months following discharge from residential treatment, and this association was not substantially reduced after adjustment for relevant prognostic variables presumed to impact outcome. Moreover, aftercare attendance during the first 12 months was not only positively associated with outcome at 12 months but also related to increased aftercare attendance during the second 12 months (i.e., 12–24 months after discharge).

Early research by Vannicelli [57] and Costello [58] empirically documented, for the first time, the importance of continuing care with alcohol-dependent patients and served an integral role in the field's apparent shift in focus from the initial treatment contact episode solely to a consideration of some form of lower intensity services following discharge from the primary phase of treatment. Together, the findings also suggest that residential programs may be best suited to place a high priority in designing and implementing systems that emphasize patient contact immediately following discharge and work to increase attendance at available continuing care options, particularly in the first 12 months after discharge. Furthermore, although increased attendance at the various forms of continuing care was associated with better outcomes in both studies, it is important to note that patients averaged only 6 continuing care visits in the second year following discharge in the Costello study. That is, it appears that the potential for a stronger, more favorable continuing care-outcome relationship may have been observed had patients participated more fully in the various continuing care program options available. However, several methodological limitations may limit the generalizability of the findings to additional populations, including those dependent on a substance other than alcohol and those for whom the initial treatment episode does not consist of residential care in the context of inpatient hospitalization.

#### 3.2.1. Community-Based Self-Help Groups

Later research conducted by the Comprehensive Assessment and Treatment Outcome Research (CATOR) group, the largest independent evaluation service for substance use treatment programs in the USA, addressed many of the limitations of earlier work. CATOR was designed to function as an independent (i.e., not federally funded, part of a governmental agency, or owned by an individual treatment provider) clinical auditor with the task of evaluating the efficacy of various levels of treatment programs in achieving abstinence from both alcohol and illicit drugs, and documenting correlates of that recovery. All patients admitted to the programs monitored by CATOR were substance-dependent; thus, patients experiencing relatively lower levels of substance use problem severity (i.e., problem drinkers and illicit drug misusers) were not included in the patient registry system. In general, the primary treatment phase for residential patients involved both group and individual therapy sessions daily and a minimum of 9 hours of treatment care per week for outpatients. The incorporation of educational and family components comprised the typical content of the primary phase of treatment. Additional services including medical and psychiatric care were also included when indicated. Continuing care (e.g., aftercare) was defined as a period of treatment involving “less intensive and tapered continuing care of weekly outpatient services for a period of months to a year or two.” Continuing care tended to focus on the provision of relapse prevention techniques and dealing with potential obstacles that patients were likely to experience early in their recovery. Although it is to be expected that many programs monitored by CATOR varied in their delivery of specific techniques, the acceptance of an abstinence-based model was nearly universal across all participating treatment sites.

The aggregate CATOR patient registry system was comprised of over 75,000 adults and 11,000 adolescents admitted to residential and outpatient treatment, and has served as the basis for several publications, innumerable presentations, and a congressional briefing (e.g., [40, 59–61]). Although CATOR represents the largest, multisite independent evaluation of substance use treatment effictiveness in the USA to date, many studies, particularly those conducted early on in the evaluation project, presented findings from subsets of the total population. For instance, one of the earliest CATOR studies reported follow-up data from a sample of 900 patients discharged from residential treatment and examined the impact of varying levels of AA attendance on 6-month abstinence rates [62]. Specifically, patients who attended one or more AA meetings per week throughout the first 6 months experienced the best outcome, with nearly three-fourths (73%) remaining abstinent at 6 months. Patients attending multiple meetings per month (but less than one or more times per week) and those attending meetings only once per month experienced somewhat lower abstinence rates at the 6-month follow-up (69% and 45%, resp.). Finally, of those patients who did not attend a single AA meeting throughout the follow-up period, only one-third remained abstinent at 6 months. Thus, the findings suggest that differential outcome expectations are likely with respect to the initial 6 months following residential treatment completion based on frequency of AA attendance. However, given that patients were followed for only 6 months after discharge, an important question not answered by Hoffmann et al. is whether the findings would have sustained themselves over a longer follow-up period.

Subsequent follow-up CATOR reports [63, 64] provided more definitive evidence of the apparent long-term benefits of AA participation for patients discharged from both residential and IOT care. In fact, regarding patients discharged from residential treatment, the observed differential regarding the rates of abstinence from alcohol based on frequency of AA attendance was substantially higher at 12 months and was even more pronounced at 24 months [64]. At the 12-month follow-up, 76% of patients who regularly attended AA meetings at least weekly were abstinent, compared to only 56% and 54% of patients who occasionally attended (i.e., those who attended multiple AA meetings per month but less than weekly) and patients who failed to attend a meeting through the duration of the follow-up, respectively. At the 24-month follow-up, three-fourths of patients who regularly attended weekly AA meetings were abstinent throughout the entire 2 years. In contrast, only half of patients who occasionally attended AA meetings and 46% of non-AA attenders were abstinent from alcohol throughout the entirety of the 24-month follow-up. Thus, the findings extended earlier work and documented that patients who attended AA following primary treatment were more likely to experience favorable outcomes with respect to abstinence and that treatment gains were sustained up to 2 years for both residential and IOT patients. Although the presented data utilized a statistical correction procedure to account for potential inflation of retrospective self-reported abstinence from alcohol due to a tendency to underreport use, further investigation is warranted to determine whether convergent rates of abstinence would have been observed for patients presenting with illicit drug use problems and, similarly, if urinalysis drug screen data were available.

Findings from a 24-month longitudinal evaluation of the accessibility and effectiveness of several outpatient SUD treatment programs provided further evidence that abstinence from both illicit drugs and alcohol varies as a function of 12-step self-help group attendance [65]. Specifically, patients admitted to outpatient treatment for a primary illicit drug problem were followed up at 6 and 24 months, although comorbid problematic alcohol use and related problems prior to admission were common among the total sample. Also noteworthy was the use of urinalysis drug screens in addition to self-reported illicit drug and alcohol use. Data regarding community-based 12-step group participation were also collected to first examine differences in abstinence rates among those patients who attended any 12-step meetings versus those who did not attend any meetings and then compare the abstinence rates from subgroups of patients classified into one of four a priori categories on the basis of their level of 12-step group participation (i.e., patients who attended meetings weekly or more frequent throughout the 24-month follow-up period, patients who discontinued 12-step participation after 6 months, patients who initiated participation in 12-step groups after 6 months, and patients who failed to attend any meetings through 24 months). Two-year outcomes revealed that nearly three-fourths (72.7%) of patients who attended any 12-step meetings reported past 6-month abstinence from illicit drugs at the 24-month follow-up, compared to only 56.0% of those patients who did not attend any 12-step meetings. Urinalysis drug screen findings confirmed the self-reported illicit drug abstinence rates. Further investigation revealed that, of those patients reporting any 12-step participation, weekly or more frequent participation was associated with an increased past 6-month abstinence rate from illicit drugs at 24 months (77.7%). Similar to illicit drug use, any participation in 12-step programs was associated with a higher past 6-month abstinence rate at 24 months with respect to alcohol relative to no participation (68.0% versus 38.8%, resp.), and weekly or more frequent participation was correlated with an increased rate of abstinence from alcohol at 24 months (74.8%).

Comparisons regarding the observed abstinence rates from the 6- to 24-month follow-up interval based on level of 12-step participation also revealed several notable findings. For instance, abstinence rates for both illicit drugs and alcohol were sustained at the second follow-up for those patients classified as “persistors” (i.e., continued to attend weekly or more frequently after the first 6 months through 24 months). Patients who discontinued 12-step participation after the first follow-up, however, experienced a significant reduction in both drug and alcohol abstinence (e.g., 85% of “dropouts” were abstinent from drugs at the first follow-up, compared to only 63% at the second follow-up). Interestingly, patients who initiated 12-step participation after the first follow-up did not experience any differences in past 6-month drug or alcohol abstinence from the first to the second follow-up. Thus, the findings from Fiorentine [65] suggest that the importance lies on treatment continuity and attendance at community-based self-help groups should be emphasized immediately following completion of the primary phase of treatment.

Together, the aforementioned studies described to this point [62–65] all documented that any participation in community-based 12-step programs (e.g., AA) was associated with increased rates of abstinence and that the magnitude of the association was similar for both illicit drug and alcohol use. Weekly or more frequent attendance at 12-step meetings was also related to more favorable outcome at both 6 and 24 months following both residential and outpatient treatment discharge. However, community-based self-help groups, such as AA, represent only one of many potential continuing care options for patients recently discharged from the primary phase of treatment. The lack of an experimental design and the failure to investigate the potential clinical utility of various continuing care alternatives available to patients may have also introduced the possibility for patient selection bias.

#### 3.2.2. Additional Continuing Care Options

In their investigation of the long-term outcomes associated with a combination of the various continuing care options available to patients, Miller and Hoffmann [16] followed a large sample of patients discharged from both residential (*n* = 6,508) and intensive-outpatient (*n* = 1,572) levels of care for 12 months. Alcohol dependence was the SUD diagnosis that predominated among both levels of care, although more than half of the total sample was dependent on a substance other than alcohol. In addition to AA, a formal outpatient aftercare program provided by the facility at which the patients completed their primary phase of treatment was available to patients. Perhaps the most notable finding was the apparent interplay between AA and formal aftercare in regard to 12-month abstinence rates following primary treatment discharge. Also of interest was the finding that both inpatients (i.e., residential) and outpatients (i.e., IOT) demonstrated comparable 12-month outcomes. In other words, irrespective of the patients' primary level of care or their extent of involvement in the two continuing care options, patients who attended either AA or the formal aftercare program provided by the treatment facility were more likely to remain abstinent than nonattenders across both levels of care. More detailed analyses revealed that less than half of the total sample (45%) who received less than 6 months of the aftercare program and did not attend AA for the entire first year remained abstinent at 12-months post-primary treatment discharge. One year of regular AA attendance in the absence of a minimum of 6-month participation in the aftercare program yielded a 12-month abstinence rate of 69%. One-year participation in the aftercare program in the absence of regular AA attendance resulted in an abstinence rate of 77%. However, patients who attended AA on a weekly basis and participated in the formal aftercare program throughout the entire 12 months following discharge from primary treatment had the best outcome, with 90% reporting past 12-month abstinence at 1 year. Overall, the findings presented by Miller and Hoffmann demonstrated several important implications for clinical practice. For instance, not only did patients discharge from both residential and IOT care benefit from some form of continuing care, but also there appears to be an additive contribution of offering a formal aftercare program in addition to AA with respect to outcome. That is, rather than offering AA alone, which has historically been the most common form of continuing care available to patients after discharge, providing patients with a menu of continuing care treatment options appears to be the better practice if long-term abstinence is desired.

Further evidence in support of the additional benefit of providing varied continuing care options following primary treatment discharge can be gleaned from two 12-month prospective studies of substance-dependent patients discharged from residential treatment programs [66, 67]. The first examined the impact of a structured cognitive-behavioral aftercare program on study outcomes relative to an unstructured program consisting of crisis counseling at the patient's request after discharge from residential treatment [67]. Main findings revealed that patients randomly allocated to the structured program experienced a fourfold increase in aftercare attendance and one-third the rate of uncontrolled substance use (e.g., consuming more than 4–6 standard drinks on a single drinking occasion or using opioids more than one time in a day) compared to the unstructured aftercare group. In the second study, patients were self-selected into one of four available continuing care options at discharge from residential treatment: (1) outpatient treatment only, (2) 12-step self-help groups only, (3) outpatient treatment in addition to 12-step self-help groups, and (4) no continuing care. Outcomes included self-reported alcohol use and additional relevant measures of psychosocial functioning. Patients who participated in both continuing care options (i.e., outpatient treatment and self-help groups) demonstrated the best 12-month outcomes (e.g., 62.5% abstinent), while those who did not obtain any form of continuing care fared the worst on all outcomes (e.g., 33.1% abstinent). Moreover, patients who had more outpatient treatment contacts, attended 12-step groups more frequently, or were more involved in 12-step activities (e.g., having a sponsor, reading the “Big Book,” working the steps) demonstrated better 12-month outcomes following discharge from residential treatment. Similar to the findings presented by Miller and Hoffman [16], the duration of treatment for patients who participated in formal outpatient programming only (i.e., in the absence of self-help group participation) was positively associated with 12-month abstinence. That is, 71.7% of patients who regularly participated (i.e., at least twice per month) in outpatient treatment for 9 months or longer were abstinent at 12 months, compared to only 37.4% and 48.9% of patients who regularly participated in outpatient treatment for 3 and 6 months, respectively. Furthermore, patients attending formal group aftercare programming on a weekly basis following inpatient treatment completion have also been found to be three times more likely to remain abstinent from alcohol at 9 months after discharge than patients who dropped out of the formal aftercare program [68].

Thus, encouraging regular attendance and participation in a combination of formal aftercare programming and self-help groups may enhance 12-month outcomes after discharge from residential or IOT care. In fact, several studies have reported similar findings regarding the relative, incremental contribution of both forms of continuing care in the prediction of various long-term posttreatment alcohol and drug use outcomes (e.g., risk for relapse), above and beyond relevant pretreatment demographic and clinical variables [69–73]. However, some evidence suggests that differential outcome expectations may be observed among specific subgroups of patients [74–76]. For instance, the clinical severity of DSM-IV (APA, [1]) alcohol dependence has been shown to impact the relative benefits of AA versus formal aftercare services among older (i.e., 65 years of age or older) patients discharged from residential, IOT, or some combination of the two levels of care [76]. Overall, the grouping of at least weekly AA attendance and 4 months or more of participation in formal aftercare programming yielded the best outcomes for both high and low clinical severity patients. However, the differentials for aftercare services, irrespective of AA attendance, were much greater for the high severity patients than for the low severity cases. In contrast, the differentials based on AA attendance, irrespective of the duration of aftercare services, were much greater for the low severity cases than they were for the high severity cases. Thus, a minimum of 4 months of formal aftercare programming appears to be more critical for alcohol-dependent patients with a higher level of clinical severity, even in the presence of regular (i.e., weekly) AA attendance.

### 3.3. Continuing Care Treatment Modality

As noted previously, considerable research has amassed to support the contention that, in general, patients clearly demonstrate favorable clinical outcomes following completion of the primary phase of treatment, irrespective of the specific theoretical orientation of the treatment provider [77–79]. There is also some evidence suggesting that differential outcomes may be expected for selecting subgroups of patients receiving continuing care based on various pretreatment demographic and clinical variables [75, 76, 80, 81]. Additional support in favor of matching continuing care services to patient characteristics has been found regarding the specific modality of the continuing care treatment [74]. Brown et al. investigated matching patient attributes to two 10-week group-based continuing care treatments among a naturalistic sample of patients recently discharged from residential treatment. Following completion of the primary phase of treatment, patients were randomized into either structured relapse prevention (i.e., a cognitive-behavioral approach in which the focus of treatment is on the identification of high-risk situations related to substance use) or a twelve-step facilitation (TSF) continuing care program (i.e., a program based on AA or NA principles). Four patient characteristics were matched to treatment: age, gender, extent of substance use, and overall psychological status. Substance use outcomes were assessed at 3 and 6 months following completion of each respective 10-week continuing care program.

Despite the finding that no differential outcomes were found for male patients at the 6-month follow-up (i.e., male patients benefited from both relapse prevention and TSF continuing care options), female patients and those reporting use of multiple substances were found to demonstrate better alcohol outcomes with TSF relative to their cohorts allocated to the relapse prevention condition. Patients with higher psychological distress at treatment entry were also able to maintain longer periods of abstinence with TSF compared to their cohorts who received relapse prevention continuing care. However, the structured relapse prevention program was found to result in better outcomes regarding the maintenance of abstinence for patients who reported lower psychological distress. Not surprisingly, random assignment that was consistent with patient preference was found to be associated with better substance use outcomes at the 6-month follow-up compared to inconsistent assignment. Thus, in the absence of multiple continuing care options, the adoption of structured TSF continuing care program appears to be a reasonable strategy and may possess a slight advantage. In contrast, a structured relapse prevention program may be suitable for patients with a lower level of overall psychological distress. When multiple continuing care options are available for patients, however, a program that is consistent with patient preference has the most potential to contribute to the overall efficacy of the program, irrespective of the modality of treatment (i.e., TSF versus relapse prevention).

Additional studies evaluating the efficacy of various conceptually distinct continuing care options in reducing substance use have produced convergent findings. In a comparison of patients randomly assigned to receive 10 weeks of either structured relapse prevention or TSF continuing care following discharge from residential treatment, both continuing care options were associated with improvement on all substance use outcomes (i.e., abstinence, severity of alcohol, and illicit drug use) and there were no between-group effects detected [82]. That is, relapse prevention and TSF were equally effective with respect to substance use outcomes. It is important to note, however, that the specific skills and topics covered by relapse prevention and TSF programs vary in the fact that relapse prevention focuses on the utilization of cognitive-behavioral processes to produce change via an individualized treatment plan, while TSF is designed to facilitate utilization of the specific principles (e.g., 12 steps) described by AA. Given the theoretical and programmatic differences in service delivery for the two group-based continuing care options, the authors also examined whether the observed effects were related to their specific hypothesized mediators. In fact, the results supported such a claim, but stronger and more consistent findings were observed in those patients who received relapse prevention as opposed to TSF. Specifically, perceptions of temptation to high-risk situations were lower and confidence in high-risk situations was higher at the end of the 10-week relapse prevention program compared to the TSF group; although these changes were not found to persist beyond the continuing care treatment phase. In other words, it appears that, although relapse prevention participation results in increased self-efficacy, this effect lasts only over the planned duration of the continuing care program and not up to the 6-month posttreatment follow-up. Overall, both continuing care regimens offered comparable benefits to substance-dependent patients and, irrespective of treatment modality, commitment to achieving the specific intervention objectives targeted was associated with favorable outcomes at 6 months. Frequency of attendance at the relapse prevention program but not TSF was also related to positive substance use outcomes at 6 months. Thus, sufficient exposure to a structured relapse prevention program appears more important to outcome compared to attendance in TSF.

Comparative continuing care studies have also evaluated the 6-month outcomes of relapse prevention versus interpersonal process groups for alcohol-dependent patients recently discharged from residential treatment [83]. Both continuing care programs were group based and consisted of eight 90-minute sessions, held weekly. The relapse prevention program was based on the social learning model of relapse and included the provision of various cognitive-behavioral techniques designed to assist patients in abstaining from substance use; specific techniques included self-monitoring of substance use, identifying high-risk drinking situations, cognitive restructuring, learning appropriate assertiveness skills, and coping with anger and urges to drink. Sessions included didactic instruction, modeling, and the use of role play to convey essential programmatic elements. Weekly behavioral or cognitive homework assignments were also included in an effort to afford the patients with an opportunity to practice the specific strategies outside of the treatment session. Conversely, the focus of the interpersonal process program was not necessarily on the attainment of abstinence from alcohol but rather on the underlying interpersonal mechanisms purported to lead to problematic alcohol use and maladaptive behavior. In addition, the interpersonal process program did not include the use of role play, cognitive restructuring, or weekly homework assignments. Overall, both continuing care programs resulted in comparable improvement on alcohol consumption, alcohol-related impairment, abstinence rates, and additional indices of alcohol use, as well as similar rates of attendance at the 6-month follow-up. Specifically, exactly half of the patients in the relapse prevention program were abstinent at 6 months, compared to 42.1% of patients in the interpersonal process program. Despite the general conclusion that both continuing care programs appear to be viable treatment modalities, several methodological limitations, most notably the particularly small sample size (*N* = 39) and the resultant inadequate power necessary to detect meaningful differences between groups, as well as the issue of poor treatment integrity in the relapse prevention group, temper the observed findings. Together, the aforementioned comparative continuing care treatment studies suggest that, in general, the specific treatment modality appears to offer little clinical value with respect to a variety of substance use outcomes.

### 3.4. Extended Continuing Care Monitoring Programs

As noted previously, substance dependence is commonly conceptualized as a cyclic, chronic condition characterized by recurrent episodes of treatment and subsequent relapse with brief periods of remission [53, 54]. Continuing care over a protracted period of time, supplemented with routine monitoring, therefore, appears to be a more viable option than a series of independent treatment episodes. One subgroup of substance-dependent patients for which this approach may prove particularly prudent involves physicians. Physicians with substance dependence represent an important population for several reasons beyond those from obvious public safety and public health perspectives. In fact, the very environment in which physicians are employed places them at elevated risk for relapse given the various high-risk situations that physicians may encounter on a routine basis in the context of performing their daily occupational responsibilities (i.e., nearly continual exposure and greater access to various substances of high abuse potential). In an effort to protect the public while also providing an opportunity for these individuals to salvage their careers and lead meaningful and productive lives, a novel form of treatment management was developed for substance-dependent physicians in recent years; Physician Health Programs (PHPs). The PHP model provides active care management, as well as routine monitoring and supervision, for physicians who have signed formal, binding contracts for participation in extended treatment—typically for a minimum of 5 years [84]. The PHPs strive to develop and maintain a close working relationship with their state medical licensing boards, and the boards often accept the care of the PHP as opposed to imposing disciplinary actions for physicians. However, an important stipulation of the contractual agreement is that a failure to adhere to the specific treatment recommendations provided by the PHP and/or evidence of a return to the use of alcohol or illicit drugs via positive urinalysis drug screen (UDS) findings will result in referral back to the licensing board for disposition.

Specifically, the extended period of PHP treatment begins with a comprehensive evaluation followed by 3 months of either residential or IOT care. A primary treatment goal for all patients is total abstinence, which is in line with the principles of AA/NA and other 12-step programs from which most PHPs operate. Physicians commonly withdraw from medical practice during the initial intensive treatment phase and upon successful completion, often return to work during the second, less-intensive phase under close supervision by the PHP. The second phase typically consists of two to three days of group outpatient therapy for 3 to 12 months; however, individual therapy is also available for patients with comorbid psychiatric or medical conditions. Although the actual time spent in formal treatment is variable due to the patient's individualized needs, inherent to the PHP model is that all patients receive active routine monitoring and care management. In addition to random UDS testing, the contractual agreement stipulates intense and ongoing treatment and compliance monitoring, as well as unscheduled work site visits or work site monitors for an extended period of time. Finally, continued participation in AA/NA or similar community-based 12-step-oriented group supports is expected of all patients after treatment.

The largest evaluation conducted to date regarding the effectiveness of this intensive and extended continuing care treatment approach involved a 5-year retrospective, intent-to-treat analysis of 904 physicians consecutively admitted to 16 state-level PHPs [84, 85]. Nearly all (88%) of the patients met diagnostic criteria for substance dependence and the remaining patients met criteria for substance abuse. Alcohol represented the primary substance of choice reported by half of the patients, followed by one-third for opioids. All patients were monitored as a standard part of their contract, described previously. Main findings revealed that approximately 9 out of every 10 patients who completed all program requirements failed to produce a single positive UDS finding during an average of 4 years of testing at a cumulative rate of 1.7 tests per month. Although not quite as marked, but still encouraging, was the finding that nearly 80% of the total sample (i.e., patients who completed the program, patients who continued to receive care following fulfillment of all contractual obligations, and patients who dropped out of treatment prematurely) was abstinent during a similar timeframe. Results also indicated that nearly three-fourths of the physicians followed were still licensed and resumed practice under supervision and monitoring with no indications of substance use or malpractice 5 to 7 years after signing their contract. Comparable findings have also been observed in previous research with physicians treated within the PHP framework (e.g., [27, 28, 67]); although these studies included smaller sample sizes and/or shorter follow-up intervals.

Interestingly, over half (55%) of the sample was formally mandated to enter the PHP by a licensing board, hospital, or other agencies; however, it is likely that the remaining patients were also mandated by families, employers, and so forth in an informal manner. Thus, preliminary findings suggest that continuing care involving intensive routine monitoring appears to represent an effective treatment option and has the potential to result in favorable long-term outcomes, at least with respect to highly motivated patients. Another important consideration of PHPs is that physicians enrolled in this form of care have significant internal and external incentives (e.g., desire to continue to practice medicine and avoid license revocation and professional disgrace) to comply with their contracted treatment and monitoring requirements. That is, although all patients, irrespective of occupation or circumstances, are likely to experience significant consequences (positive or negative) based on whether or not they fully comply with program requirements and recommendations in the context of primary and secondary treatment, the implications are arguably much greater for physicians than they may be for other patients. However, the positive findings do suffice to demonstrate that inclusion of similar key programmatic elements such as contingency management, routine UDS testing, and linkage with community-based 12-step programs may translate to improved mainstream continuing care treatment efforts with additional nonphysician populations.

### 3.5. Enhancement of Treatment Engagement in Continuing Care Services

The findings from our review of the vast substance use treatment literature all point to the value of some form of continuing care following the primary phase of treatment. Thus, although it is of paramount importance that patients receive some form of lower intensity care following treatment discharge, irrespective of the initial level of care, perhaps the greater issue is how best to engage and motivate patients so that they will follow through on any continuing care regimen that is recommended following completion of the initial phase of treatment. Given the incremental value of continuing care to successful clinical outcomes, several studies have examined the potential utility of various strategies designed to engage patients in continuing care participation to determine whether there is any value in vigorously encouraging patients to participate in continuing care activities, and what methods may prove most prudent in terms of increasing adherence.

Early research in the area of continuing care treatment engagement [86] compared the effect of a brief (i.e., 20 minutes) orientation session to a minimal treatment condition on outpatient group therapy participation among a sample of substance-dependent patients recently discharged from residential treatment. The orientation session consisted of an individual meeting with a facilitator of the group, in which encouragement and adequate rationale regarding the importance of continuing care were provided. Patients in the brief orientation condition were also asked to sign a continuing care participation contract. Patients in the minimal interaction condition watched a generic video in which the content focused on motivation to reach goals. Patients who received the continuing care orientation session were more likely to attend continuing care treatment relative to those who received the minimal interaction session (70% versus 40%, resp.). The brief orientation session was also associated with increased attendance at outpatient sessions.

Although a brief orientation session including encouragement and adherence contracts appears to be an effective method to increase continuing care attendance, later work investigated whether feedback and prompts would further enhance the clinical utility of such components among a sample of substance-dependent patients recently discharged from residential or IOT care [87]. Patients were randomly assigned to receive either attendance feedback and prompts to attend the recommended continuing care program or no feedback and no prompts. Findings revealed that patients who received the feedback and prompts were significantly more likely to initiate the continuing care program and attended more weekly outpatient group therapy sessions. The impact of social reinforcement in addition to a standard orientation session on continuing care attendance has also been examined [88]. At the 6-month follow-up, patients who received social reinforcement were significantly more likely to be abstinent than those patients who received the standard orientation session alone (76% versus 40%, resp.) after discharge from residential treatment. Patients allocated to the social reinforcement group were also found to evince better long-term continuing care attendance relative to the standard orientation group.

Finally, in theory, considering that many patients are likely to encounter a variety of problems following primary treatment discharge, telephone follow-up initiated by clinical staff may represent a feasible and viable option for exchanging information, providing advice, recognizing complications and barriers to recovery early, and providing reassurance to patients throughout the continuing care treatment phase. In fact, several studies have tested the effect of adding routine telephone-based follow-up contacts to standard continuing care practices among substance-dependent patients (e.g., [89–93]), and the general consensus is that this strategy is associated with improved clinical outcomes. For instance, McKay et al. [91] tested the effect of adding up to 18 months of telephone continuing care to intensive-outpatient treatment on outcomes among a sample of alcohol-dependent patients following three weeks of intensive-outpatient treatment. Findings revealed that the combination of telephone continuing care and intensive-outpatient treatment improved alcohol use outcomes relative to intensive-outpatient programming alone. Together, these findings suggest that several low-cost strategies designed to enhance patient engagement in continuing care may prove useful with respect to increasing adherence, which in turn, may increase the likelihood of achieving positive long-term clinical outcomes.

## 4. Conclusions and Recommendations for Clinical Practice

Substance use and SUDs represent major public health concerns and are associated with a variety of unfavorable outcomes including increased health care utilization, decreased work productivity, and substantial economic burden to both individual patients and society in general. A viable option to offset the significant social, medical, psychological, and economic costs associated with SUDs is substance use treatment. In fact, it is well established that appropriate treatment placement and completion have been shown to improve a wide range of areas related to patient functioning, irrespective of the specific theoretical orientation of the clinician, professional background and personal substance use history of the clinician, and in many instances, level of care received. However, substance use treatment efforts are increasingly being viewed and contextualized within a similar disease management framework, much like that of other chronic medical conditions (e.g., diabetes, hypertension), in which the incorporation of some form of lower intensity continuing care services delivered in the context of outpatient treatment after the primary treatment phase (e.g., residential). In other words, it is imperative that both treatment providers and policymakers support the adoption of a continuing care approach in the treatment of substance dependence and consider treatment not from an acute, but from a chronic care perspective.

The commonly held view that continuing care attendance improves the posttreatment prognosis of alcohol-dependent patients stems from early research by Costello [58] and Vannicelli [57]. Considerable evidence in the form of systematic reviews and controlled outcome studies has since found that both alcohol- and drug-dependent patients, as well as patients discharged from both inpatient and outpatient treatment, appear to benefit from continuing care services (e.g., [94]). Regarding the first part of the main objective of the present review, to identify what “works” (i.e., the specific treatment components that comprise an effective continuing care model), the research literature suggests that offering a combination of services after discharge from primary treatment may represent the best practice if long-term abstinence is to be expected (e.g., [16, 66]). That is, there appears to be an additive contribution of offering a formal outpatient aftercare program in addition to community-based self-help groups with respect to outcome, rather than simply encouraging patient attendance at AA/NA meetings alone. In addition, the frequency of continuing care attendance, whether it is in the form of community-based self-help groups such as AA or more formal outpatient aftercare programming, during the initial 12 months following primary treatment completion has also been shown to be positively related to the likelihood of abstinence.

Numerous comparative continuing care treatment studies have also found that, in general, the specific treatment modality (e.g., relapse prevention, 12-step) appears to offer little clinical value with respect to a variety of substance use outcomes (e.g., [95]). Given the overarching goal of relapse prevention programming (i.e., long-term maintenance of treatment gains), such an approach may be particularly well suited for implementation in continuing care contexts. Greater affiliation with AA during the continuing care phase of treatment, however, has been found to predict better long-term outcomes [96]. Similarly, patients who endorsed a goal of absolute abstinence on entering continuing care have fared better from group relapse prevention programming than 12-step group therapy [95]. Thus, although some evidence suggests that various specific continuing care treatment modalities have the potential to enhance outcomes with specific subgroups of patients based on specific individual difference and pretreatment demographic characteristics, a modality that is consistent with patient preference appears to possess the most value in terms of contributing to the overall efficacy of the program.

Although the most common continuing care approach has traditionally involved the separation of SUD treatment services into distinct phases, the use of alternative adaptive continuing care approaches has become increasingly more common in recent years. In fact, accumulating evidence and recent developments in the long-term care of substance-dependent patients suggest that PHPs and additional alternative adaptive treatment approaches may serve as pragmatic treatment options [84, 85]. Such approaches are designed to retain patients in treatment for an extended period of time and involve the modification of service delivery and the accompanying intensity of care based on patient response via extended routine monitoring. Preliminary findings suggest that continuing care involving active routine monitoring appears to show promise as an effective strategy and has the potential to result in favorable long-term outcomes for patients with higher perceived levels of motivation. Additional research, however, regarding the efficacy of PHPs and similar adaptive continuing care approaches in the long-term treatment of substance dependence among additional nonphysician populations, is clearly warranted.

In response to the second part of the main objective of the present review, to identify when “enough,” is “enough,” a precise answer is not evident and it is not likely to be simple given the complexity of patient needs that most treatment programs are expected to address, coupled with the fact that individual treatment programs are diverse and vary widely in the specific services provided. Generally speaking, however, our review of the vast continuing care treatment literature clearly points to the value of at least 3 months or longer of continuing care services. That is, prior to the 3-month mark, little to no incremental net benefit in terms of abstinence rates is typically observed when patients receive 1 or 2 months of continuing care relative to patients who do not receive some form of continuing care. Similarly, there appears to be a trend once patients receive continuing care for a period lasting at least 6 months. Although not quite as marked, the same threshold effect can be found at 9 and 12 months. Finally, 2-year outcomes are virtually identical to those seen at 1 year. Thus, irrespective of the primary treatment episode, continuing care over a protracted period for a minimum of 12 months appears to be a requisite if abstinence rates above roughly 65% are desired. This apparent positive trend involving duration of services and abstinence is relevant from not only a clinical standpoint, but also an economical one as well. That is, there appear to be levels of care too low to reach the targeted outcome, while the delivery of services at the same intensity level beyond an identified point in time may produce relatively little clinical benefit. The latter is particularly salient given the cost-conscious times in which substance use treatment operates and the fact that the provision of intensive services beyond this point may be considered a misuse of already limited resources.

It is important to note that the present review of the continuing care treatment outcomes literature should be considered in light of several limitations that may limit the generalizability of the discussed findings. For instance, many of the studies included relatively small sample sizes and/or brief follow-up periods. The limitation pertaining to sample size is particularly salient given that small sample sizes have the potential to result in marginally significant effect sizes. In the instance of limited observational or follow-up periods, it remains unclear if the reported findings would have sustained themselves over a longer follow-up period, or conversely, if nonsignificant findings would have been associated with favorable outcomes at a later point in time. Also noteworthy is that many outcome studies vary considerably in their measurement of abstinence, which makes comparisons across studies difficult. For example, at 1 year, the observed abstinence rate following discharge from the primary phase of treatment may include only the past 30 days or the past 6 months at the 12-month mark for some studies, while others may examine the patients' abstinence throughout the entire 12-month period.

Additionally, although the associations between participation in some form of continuing care services following discharge from the primary phase of treatment and favorable long-term clinical outcomes are quite strong, the cross-sectional nature of the reported data for many studies limits the ability to determine causality. Thus, many of the findings presented here are only suggestive and may have been confounded by such issues as patient selection bias, among others. As such, the studies reviewed here and the reported outcomes may be more appropriately conceptualized as an important first step in identifying the value of continuing care in the achievement of long-term abstinence from alcohol or illicit drugs. More definitive evidence in the form of randomized controlled trials in which clearly described continuing care treatment programs and comparison or control groups of adequate size are followed over a sufficiently long period following primary treatment completion is an essential next step. However, observational studies of naturalistic treatment settings, in which patients exercise a considerable degree of control over their treatment, have the potential to offer important evidence about continuing care treatment efficacy not readily available from randomized controlled trials.

Furthermore, in the case of a study's failure to include a formal control group for comparison, it remains difficult to separate treatment effects from those of other relevant factors known to significantly impact outcomes such as the individual circumstances of the patients presenting for treatment. Likewise, the collection of data on planned duration, intensity, and content of continuing care programs is equally important for future work in an effort to clearly differentiate between treatment and motivation effects. Further consideration of the role of various individual difference variables and examination of potential mediators and moderators is also a requisite for future investigations. Finally, as is the case with the use of all self-report follow-up data, the possibility remains that response and recall bias may have been introduced given the nature of such a method. Similarly, many studies relied on self-reported, retrospective accounts of substance use and use-related problems, and urinalysis drug screens were often not included as a standard part of the clinical protocol. Thus, it was not possible to routinely confirm the veracity of self-reported abstinence rates or verify that other types of substances were not being used. Utilization of multiple informants and multiple methods such as biological verification of substance use is essential to provide objective evidence regarding the detection of the presence or absence of specified substances.

In light of these methodological limitations, the data clearly indicate that the duration of continuing care should extend for a minimum of 3 to 6 months if individual patients and all relevant stakeholders hope to achieve a reasonable expectation of robust recovery. Ideally, some contact over a 12-month continuum yields a rational balance between investment and outcome. To conclude our review of the literature and discussion of the key programmatic elements and best practices essential to the planning and implementation of an adequate continuing care treatment model, several recommended strategies may be considered. First, it is of paramount importance that primary treatment programs provide the patient with sufficient education regarding available continuing care options, including pharmacotherapy if applicable (e.g., naltrexone, buprenorphine, methadone maintenance), in a timely manner prior to treatment completion. Second, the provision of some form of lower-intensity continuing care services as an in-house adjunct to the treatment program which incorporate some of the earlier program elements while also offering new elements such as community-based self-help groups is recommended. Third, should the primary treatment program not have such services available, the program is advised to link patients with relevant supportive services in the community upon discharge in an effort to ensure increased communication and continuity of care. Fourth, the treatment program should include appropriate follow-up procedures over an extended period of time, during which the program regularly follows up with the patient at designated intervals after discharge (e.g., 30 days, 6 months, 12 months) for a minimum of 12 months. In an effort to minimize the burden to both patients and providers, the frequency and intensity of treatment may also be modified accordingly based on the patients' response to the indicated treatment plan. Finally, beyond the implementation and inclusion of an adequate follow-up period, treatment providers must include some form of an outcome monitoring system, in which relevant clinical information related not only to the patients' self-reported substance use but also to relevant changes in functioning that may increase their potential for relapse is obtained at regular, predetermined intervals.
